# Antimicrobial Properties and Mechanism of Action of Some Plant Extracts Against Food Pathogens and Spoilage Microorganisms

**DOI:** 10.3389/fmicb.2018.01639

**Published:** 2018-07-24

**Authors:** Faraja D. Gonelimali, Jiheng Lin, Wenhua Miao, Jinghu Xuan, Fedrick Charles, Meiling Chen, Shaimaa R. Hatab

**Affiliations:** ^1^College of Food Science and Pharmaceutics, Zhejiang Ocean University, Zhoushan, China; ^2^Department of Food Science and Technology, College of Agricultural Science and Fisheries Technology, University of Dar es Salaam, Dar es Salaam, Tanzania; ^3^Zhoushan Institute of Food and Drug Inspection, Zhoushan, China; ^4^Faculty of Environmental Agricultural Science, Arish University, North Sinai, Egypt

**Keywords:** plant extract, ultrasound-assisted extraction, antimicrobial properties, internal pH (pH_int_), membrane potential, spoilage, pathogenic microorganism

## Abstract

This work aims to evaluate the antimicrobial potential of ethanolic and water extracts of roselle (*Hibiscus sabdariffa)*, rosemary (*Rosmarinus officinalis)*, clove (*Syzygium aromaticum*), and thyme (*Thymus vulgaris*) on some food pathogens and spoilage microorganisms. Agar well diffusion method has been used to determine the antimicrobial activities and minimum inhibitory concentrations (MIC) of different plant extracts against Gram-positive bacteria (*Bacillus cereus, Staphylococcus aureus*), Gram-negative bacteria (*Escherichia coli, Salmonella enteritidis, Vibrio parahaemolyticus*, and *Pseudomonas aeruginosa*), and one fungus (*Candida albicans*). The extracts exhibited both antibacterial and antifungal activities against tested microorganisms. Ethanolic roselle extract showed significant antibacterial activity (*P* < 0.05) against all tested bacterial strains, while no inhibitory effect on *Candida albicans* (CA) was observed. Only the ethanolic extracts of clove and thyme showed antifungal effects against CA with inhibition zones ranging from 25.2 ± 1.4 to 15.8 ± 1.2 mm, respectively. *Bacillus cereus* (BC) appears to be the most sensitive strain to the aqueous extract of clove with a MIC of 0.315%. To enhance our understanding of antimicrobial activity mechanism of plant extracts, the changes in internal pH (pH_int_), and membrane potential were measured in *Staphylococcus aureus* (SA) and *Escherichia coli* (EC) cells after exposure to the plant extracts. The results indicated that the plant extracts significantly affected the cell membrane of Gram-positive and Gram-negative bacteria, as demonstrated by the decline in pH_int_ as well as cell membrane hyperpolarization. In conclusion, plant extracts are of great value as natural antimicrobials and can use safely as food preservatives.

## Introduction

Globally, food spoilage caused by microorganisms still widely affects all types of food and causes food waste and loss, even in developed countries. It has been estimated that the yearly losses of global food reach up to 40% due to various factors including spoilage by microorganisms (Gustavsson et al., 2011). Bacteria, yeast, and molds are the common types of microorganisms responsible for the spoilage of a considerable number of food and food products (Lianou et al., 2016). Once these microorganisms reach food products, they grow by utilizing the nutrients and produce metabolites that cause food spoilage (Parlapani et al., 2017). Foodborne disease is another pervasive food safety problem caused by consumption of contaminated food products, which has been a significant safety concern to public health (Azziz-Baumgartner et al., 2005; Kirk et al., 2017).

Microorganisms are available naturally in the surrounding environment; therefore they can easily reach food during harvesting, slaughtering, processing, and packaging (Hatab et al., 2016). These microorganisms can survive under adverse conditions used in the food preservation such as low temperature, modified atmosphere packaging, vacuum packaging, as well as resist conventional pasteurization (Dimitrijević et al., 2007; Provincial et al., 2013; Saraiva et al., 2016; Säde et al., 2017). Thus, there is a considerable concern among consumers regarding the risk of using synthetic additives for human health, that led to decrease the use of these chemicals in food preservation (Gyawali and Ibrahim, 2014; Caleja et al., 2016; Kalem et al., 2017). Therefore, new eco-friendly methodologies are required to reduce the growth of pathogenic bacteria and prolong the shelf-life of food products, without using chemical preservatives. Recently, many researchers investigated the possible utilization of some plant extracts as effective natural preservatives (Fernández-López et al., 2005; Suppakul et al., 2016; Clarke et al., 2017). Traditionally, the crude extracts of different parts of medical plants, including root, stem, flower, fruit, and twigs, were widely used for treatments of some human diseases (Khan et al., 2013). Medicinal plants contain several phytochemicals such as flavonoids, alkaloids, tannins, and terpenoids, which possess antimicrobial and antioxidant properties (Talib and Mahasneh, 2010). The antimicrobial activities of some plant species have been widely researched. For example, the crude extracts of cinnamon, garlic, basil, curry, ginger, sage, mustard, and other herbs exhibit antimicrobial properties against a wide range of Gram-positive and Gram-negative bacteria (Alzoreky and Nakahara, 2003; Castro et al., 2008). In addition, it has been reported that the extracts from Chinese chives and cassia can effectively reduce the growth of *Escherichia coli* and other bacteria during storage of meat, juices, and milk (Mau et al., 2001). In a similar study, Doddanna et al. (2013) investigated the effect of some plant extracts on the growth of *Candida albicans*, the results indicated that the alcoholic extract of curry leaves effectively inhibit the growth of *C. albicans* with 24.05 ± 0.07 after 48 h. Moreover, Nzeako et al. (2006) reported that thyme oil extract could decrease the growth of *C. albicans* and *Pseudomonas aeruginosa*.

The understanding of the mechanism of antimicrobial action of medicinal plants extracts is the first step in the optimal utilization of these extracts as natural antimicrobial agents to extend the shelf-life and maintain the food quality. With this goal, this work seeks (1) to compare between two extraction methods namely conventional extraction and ultrasound techniques (2) to examine the antimicrobial activity of ethanolic and water extracts of roselle (*Hibiscus sabdariffa)*, rosemary (*Rosmarinus officinalis*), clove (*Syzygium aromaticum*) as well as thyme (*Thymus vulgaris*) against seven common food pathogens and spoilage microorganisms, (3) to understand the mechanisms of action of tested plant extracts with respect to the potential disruption in the membrane of microorganisms and changes in cytoplasmic pH (pH_int_).

## Materials and Methods

### Extraction

In this work, four plants were selected based on their traditional usage as folk medicine. The plants were purchased, in dried form, from the local market in Zhoushan, China. **Table 1** shown the plant species and the parts evaluated in this study. Two solvents (water and ethanol), and two extraction methods (conventional extraction and ultrasound techniques) were used to evaluate their effect on the extraction yield.

**Table 1:** Plant species used in this study and their extraction yield percentage by conventional and ultrasound method.

Plant species	Family	Common name	Plant part used	Country of origin	Extraction yield (%)			
					Water		Ethanol	
					
					CM^∗^	UM	CM	UM
*Hibiscus sabdariffa*	Mallows	Roselle	Flower	China	38.67 ± 0.96_a_	62.50 ± 0.51_b_	21.00 ± 0.90_a_	22.22 ± 0.45_a_
*Syzygium aromaticum*	Myrtaceae	Clove	Flower	Indonesia	19.17 ± 1.90_a_	32.16 ± 0.21_b_	16.67 ± 1.17_a_	20.00 ± 0.55_b_
*Rosmarinus officinalis*	Lamiaceae	Rosemary	leaves	Germany	17.67 ± 0.84_a_	25.00 ± 0.48_b_	14.68 ± 1.50_a_	12.14 ± 0.36_a_
*Thymus vulgaris*	Lamiaceae	Thyme	leaves	Germany	17.00 ± 1.00_a_	21.95 ± 0.89_b_	12.00 ± 1.00_a_	15.85 ± 0.34_b_

*Values are mean of triplicate determination (n = 3) ± standard deviations. Lowercase letters indicate significant differences (P < 0.05) between different extraction methods using the same solvent, where the row was split into two, the first two subtables were compared together and the last two subtables were compared together. ^∗^CM refers to Conventional method, while UM means Ultrasound method.*

For conventional extraction, 20 g powder of each tested plant material (**Table 1**) was soaked in 180 ml of distilled water in a round bottom flask and heated for 30 min at 90°C, before the overnight incubation at 37°C, and 150 rpm in a shaking incubator (Xu et al., 2008). Similarly, 10 g powder of each tested plant material was mixed with ethanol (9:1) separately in round bottom flasks and incubated at 37°C and 150 rpm for overnight. Liquid extracts obtained were separated from the solid residue by filtration using Whatman No. 1 filter, and then concentrated using a rotary evaporator (EYELA N-1100, China).

For ultrasound techniques, 10 g powder of each tested plant material was mixed with 180 ml of distilled water, or ethanol (9:1) separately in beakers. Each beaker was placed in an ultrasonic bath (KUDOS, SK8210HP, China) for 30 min at 53 kHz. Afterward, the two beakers were transferred to incubator shaker and kept for overnight at 37°C and 150 rpm. The supernatant was similarly processed as described in conventional extraction to get dried extracts of selected plants as described by (Dhanani et al., 2017). Dried extracts were dissolved in 10% DMSO for ethanolic extracts or in distal water for water extracts (Gurnani et al., 2016). The final concentration of different extracts was 20% w/v. The crude extract was then stored at −20°C for further study. The extraction yield of selected plants has been calculated by the following equation (Felhi et al., 2017):

$$\text{Yield}(\%)=(X_{1}*100)/X_{0}$$

Where *X_*1*_* refers to the weight of extract after evaporation of solvent and *X_*0*_* refers to the dry weight of the plant powder before extraction.

### Preparation of Inoculum

The antimicrobial properties of plant extracts were tested against Gram-positive bacteria [*Bacillus cereus* 10451 (**BC**), *Staphylococcus aureus* 10786 (**SA**)], Gram-negative bacteria [*Escherichia coli* GIM1.708 (**EC**), *Salmonella enteritidis*10982 (**SE**), *Vibrio parahaemolyticus* 17802 (**VP**), and *Pseudomonas aeruginosa* (B) 10104 (**PA**)], as well as one pathogenic fungus [*Candida albicans* (F) 98001 (**CA**)]. SA, BC, and SE were purchased from China Center for Industrial Culture Collection (CICC; Beijing, China), while EC was provided by Microbial Culture Collection Center of Guangdong (GIMCC; Guangdong, China). VP was purchased from American Type Culture Collection (ATCC), while PA and pathogenic fungus CA were obtained from National Centre for Medical culture collection (CMCC). The Gram-positive and Gram-negative bacteria were pre-cultured in Mueller Hinton broth (MHB) overnight in a rotary shaker at 37°C. Afterward, each strain was adjusted at a concentration of 10^8^ cells/ml using 0.5 McFarland standard (Bhalodia and Shukla, 2011). The fungal inoculum was prepared from the 48 h culture of fungal isolates in Potato dextrose broth (PDB) (Nisha et al., 2010). The spectrophotometer (A_595_ nm) has been used to adjust the spore density of fungus at a final concentration of 10^6^ spores/ml.

### Antimicrobial Screening

Agar well diffusion method was used to screen the antibacterial and antifungal activities of different solvent extracts as displayed by (Daoud et al., 2015). One ml of fresh bacterial or fungi culture was pipetted in the center of sterile Petri dish. Molten cooled Muller Hi (PDA) for fungi was then poured into the Petri dish containing the inoculum and mixed well. Upon solidification, wells were made using a sterile cork borer (6 mm in diameter) into agar plates containing inoculums. Then, 100 μl of each extract (20% w/v) was added to respective wells. The concentration of extracts (20% w/v) has been selected based on our pre-experiments, and previous literature. The plates were placed in the refrigerator for 30 min to let the extracts diffusion well into the agar. Then, the plates were incubated at 37°C for 18 h. Antimicrobial activity was detected by measuring the zone of inhibition (including the wells diameter) appeared after the incubation period. DMSO at a concentration of 10% was employed as a negative control.

### Determination of Minimum Inhibitory Concentrations

All tested extracts exhibited antimicrobial activity at a concentration of 20% (w/v). Therefore, this concentration was manipulated to determine their minimum inhibitory concentrations (MIC) using agar well diffusion method, and to evaluate their effectiveness in controlling food pathogens and spoilage microorganisms (Mostafa et al., 2018). Different concentrations 10, 5, 2.5, and 1.25% were prepared by two-fold serial dilution. 1 ml of each prepared inoculum was pipetted into sterile Petri dishes followed by the addition of molten agar and mixed well. Then, four wells were made on each plate, and 100 μl of 10, 5, 2.5, and 1.25% of each extract was transferred to the respective wells. Plates were kept in the refrigerator for 30 min and then incubated at 37°C for 18 h. The MIC was considered as the lowest concentration which inhibited the growth of the respective microorganisms. All assays were performed in triplicate. DMSO was served as a control for ethanolic extracts and distilled water was used as a control for water extracts.

### Determination of Cytoplasmic pH (pH_int_)

The two strains (SA and EC) have been chosen as an example of Gram-positive and Gram-negative bacteria to study the antibacterial mechanism of plant extracts. The changes in microbial cell cytoplasmic pH (pH_int_) was investigated as an indicator of the antimicrobial mechanism of the plant extracts. In our experiment, the fluorescent probe employed was 59′69′ carboxyfluorescein diacetate succinimidyl ester (CFDA-SE). Cytoplasmic pH (pH_int_) was carried out as described by (Molina-Gutierrez et al., 2002; Sanchez et al., 2010) with slight modifications. Briefly, EC and SA bacterial cells were cultured in nutrient broth at 37°C, 128 rpm for 24 h, followed by subculturing at the same conditions for 3 h. Cells were harvested by centrifugation at 11200 × *g*, 4°C for 5 min, washed twice using potassium phosphate (PP) buffer (50 Mm, pH 7.0) and resuspended in the PP buffer. The number of cells was adjusted to 10^8^ cells/ml by using 0.5 McFarland standard. Afterward, the bacterial cells were incubated at 37°C, 128 rpm for 30 min in the presence of 1.0 μM CFDA SE dye. The cells were centrifuged at 11200 × *g*, for 5 min and resuspended in PP buffer (pH 7), followed by addition of 10 mM glucose solution and incubated for an additional 30 min at 37°C. The cells were centrifuged at 11200 × *g* for 5 min, washed once and resuspended in PP buffer. Stained cells were then aliquoted into equal volumes for the control and treatment groups and the cell-free filtrate. One ml of plant extracts with a final concentration of 20% (w/v), were added to 1 ml of bacteria or cell-free filtrate. Fluorescence intensities were determined after 10 min by fluorescence spectrophotometer (HITACHI 4500, Japan), using excitation wavelengths of 490 nm and emission wavelengths of 520 nm. While the excitation slits widths were 5 nm and the emission slits widths were 10 nm. For the control samples and bacteria free filtrate, 1 ml of PP buffer (50 mM, pH 7.0) was added to 1 ml of bacteria. Fluorescence for bacterial cells was determined by subtracting fluorescence of the respective cell-free filtrate from the treated or control groups.

### Determination of Membrane Potential Disruption

DiBAC4(3) dye was used as described by (Sanchez et al., 2010; Clementi et al., 2014) to determine the interruption of the cell membrane. Briefly, EC and SA bacterial cells were grown in nutrient broth at 37°C, 128 rpm for 3 h. After harvesting by centrifugation at 12000 × *g* for 5 min, cells were washed using PP buffer (50 mM, pH 7.0) and resuspended in the PP buffer to about 10^8^ cells/ml using 0.5 McFarland standard. After that, 1.0 μM membrane potential-sensitive fluorescence probe DiBAC_4_ (3) was added and incubated for 30 min.

Stained cells were aliquoted into control, treatment groups and cell-free extracts. 1 ml of plant extract at a concentration of 20% was added to 1 ml of bacteria or cell-free filtrate. For control groups, 1 ml of PP buffer was added to 1 ml of bacteria or cell-free filtrate. Fluorescence intensities were determined by fluorescence spectrophotometer (HITACHI 4500), using an excitation wavelength of 492 nm and an emission wavelength of 518 nm. While the excitation slits widths were 5 nm and the emission slits widths were 10 nm, at room temperature 25°C. Background fluorescence resulting from the extracts added to the medium was determined.

### Statistical Analysis

The data represent mean of three replicates ± standard deviation (SD). Results were subjected to multiway analysis of variance, and the mean comparisons were performed by Tukey’s multiple range test using SPSS version 20.0 (Statistical Package for the Social Sciences, Inc., Chicago, IL, United States). Differences between means were considered significant at *p*-value < 0.05.

## Results and Discussion

**Table 1** summarized the extraction yield of each tested plant, which prepared by conventional or ultrasound method using water/ethanol. The extraction yield obtained by conventional method showed low percentage yield (**Table 1**) compared to those using ultrasound method. In ultrasound method, the yield of water extracts for all tested plants was much higher (*p* < 0.05) than ethanolic extracts (**Table 1**). The water extract of roselle (62.50 ± 0.51%) represented the highest yield among plant extracts followed by water extract of clove (32.16 ± 0.21%). Similarly, for ethanolic extract, the highest yield (22.22 ± 0.45%) was achieved with of roselle extract followed by clove extract (20.00 ± 0.55%), while, the lowest extract yield was obtained with thyme (15.85%) (**Table 1**). It has been reported previously that the water extract of different plants usually yields significantly higher amounts compared to ethanolic extracts of same plants (Caleja et al., 2016). This may be due to using a high temperature for 30 min during extraction and also to the higher polarity of water (Dhanani et al., 2017). In addition, the utilization of vibrations to rupture plant cell walls, resulted in releasing of compounds and molecules into the solvent (Tiwari, 2015). In this method, the thermal treatment is not applied, which helps protect the functional particles and increasing the recovered materials from the sample (Altemimi et al., 2016). It is highly recommended to use ultrasound method for the extraction of compounds from various sources and for different uses (Grassino et al., 2016).

**FIGURE 1:**
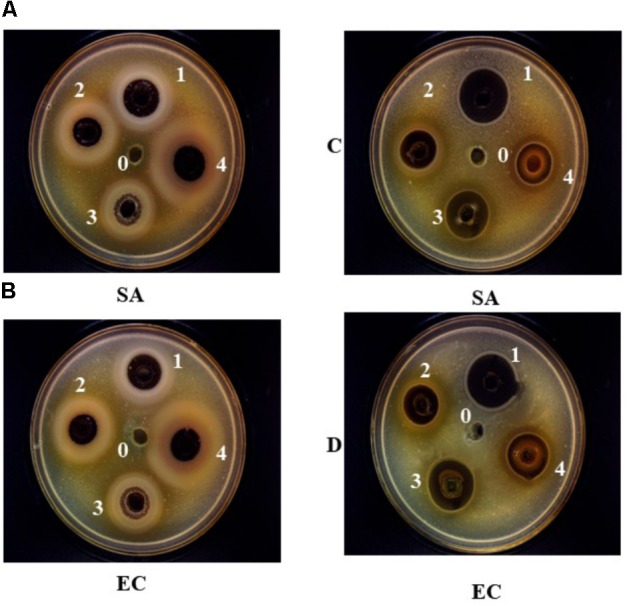
The inhibition zone (mm) of aqueous **(A,B)** and ethanolic extracts **(C,D)** of Roselle (1), Clove (2), Rosemary (3), and Thyme (4) against *Escherichia coli* (EC) and *Staphylococcus aureus* (SA), at concentration of 20% (w/v). (0) represent the negative control, 10% v/v DMSO for ethanolic extracts, and distilled water for aqueous extracts.

The antimicrobial properties of ethanolic and aqueous extracts of roselle, clove, thyme, and rosemary at a concentration of 20% against BC, EC, SA, SE, VP, PA, and CA, have been assessed in this study. The results revealed that the ethanolic and water extract of selected plants are efficiently suppressing the growth of food pathogens and spoilage microorganisms with variable potency. As stated in **Table 2** and **Figure 1**, ethanolic extract of roselle had the maximum zone of inhibition against PA (23.4 ± 1.4 mm), whereas water extract of roselle showed a maximum zone of inhibition against BC (17.0 ± 1.1 mm). The ethanolic extract of rosemary exhibited inhibitory effect against four of the pathogenic strains (EC, SE, BC, and SA) while aqueous extract of rosemary was effective against three strains only (EC, BC, and SA). In the antifungal analysis, just ethanolic extract of clove and thyme had valuable results against CA with inhibition zone (25.2 ± 1.4, and 15.8 ± 1.2), respectively. It has been reported previously that the extracts from several plants such as oregano, cumin, cinnamon, sage, and other spices possessed significant (*P* < 0.05) antibacterial and antifungal activities against wide range of food spoilage bacteria (Gram-positive and Gram-negative), as well as yeast and mold (Nassan et al., 2015; Liu et al., 2017). The antibacterial activities of ethanol extract from five plants against *Listeria monocytogenes*, SA, and SE in raw pork by counting bacterial enumeration were investigated, and the results confirmed that fewest colonies of tested bacteria were observed with clove extract (Liu et al., 2017). Burt (2004) demonstrated that the antimicrobial properties of thyme are owing to its content of thymol that could bind to membrane proteins by hydrophobic bonding and hydrogen bonding, and thus changing the permeability of the membranes. Our data confirmed that rosemary and roselle’s extracts have no antifungal activity against CA strain.

**Table 2:** Antimicrobial activity of plant extracts against seven microorganisms.

Test Strains^∗^	Zone of inhibition (mm)^a^							
	Roselle		Clove		Rosemary		Thyme	
	Ethanol	Water	Ethanol	Water	Ethanol	Water	Ethanol	Water
EC	21.1 + 1.3_a_	15.6 + 1.2_b,c_	17.4 + 0.8_b_	13.2 + 1.6_c,d,e_	21.1 + 0.9_a_	12.5 + 0.7_d_	15.9 + 0.3_b,e_	12.2 + 0.7_d,f_
VP	20.3 + 1.8_a_	15.9 + 1.7_a,b_	14.7 + 2.0_b_	13.1 + 1.8_b,c_	N	N	13.9 + 1.3_b,e_	14.3 + 0.1_b,f_
PA	23.4 + 1.4_a_	13.9 + 1.9_b,c_	17.0 + 0.5_b_	13.2 + 1.4_c_	N	N	N	N
SE	20.2 + 1.7_a_	14.0 + 1.9_b_	15.1 + 1.4_b_	12.2 + 1.1_b_	20.7 + 1.2_a_	N	N	11.8 + 1.4_b_
BC	22.2 + 0.8_a_	17.0 + 1.1_b,d,e,q_	18.2 + 3.2_a,b,e,q_	15.1 + 0.9_b,c_	19.8 + 0.8_a,d,f_	13.9 + 1.2_c,e_	17.3 + 0.7_b,e,f,q_	13.8 + 1.1_c,q_
SA	21.5 + 2.1_a_	15.7 + 1.0_b,d,q,i_	16.7 + 1.0_b,c,e_	13.6 + 1.3_d,q,i_	19.8 + 0.4_a,e,h_	12.7 + 0.4_d,f_	16.3 + 1.0_c,q,h_	12.5 + 1.4_*f,i*_
CA	N	N	25.2 + 1.4_b_	N	N	N	15.8 + 1.2_c_	N

*^a^Values are means of triplicate determination (n = 3) ± standard deviations. N, no zone of inhibition was found. ^∗^EC, Escherichia coli; VP, Vibrio parahaemolyticus; PA, Pseudomonas aeruginosa; SE, Salmonella enteritidis; BC, Bacillus cereus; SA, Staphylococcus aureus; CA, Candida albicans. Lowercase letters in the same row indicate significant differences (P < 0.05).*

This study supports previous findings in the literature that the antimicrobial activities have a direct relation to increasing the extracts concentration (%) (Bhalodia and Shukla, 2011). Significant antimicrobial effects, expressed as MIC of each plant extract against test microorganism is given in **Table 3**. The data revealed variability in the MIC among plant extracts, the lowest MIC values (0.313, 0.625%) were exhibited by water extracts of clove and roselle against BC, respectively, as well as the ethanol extracts of clove (0.625%) against VP. As put forward by Mostafa et al. (2018), that the difference in MIC of plant extracts is due to variation in their chemical constituents and volatile nature of their components. In general, the ethanolic extracts had lower MIC values than most of the corresponding aqueous extracts. This lends support to previous findings in the literature that alcoholic extracts display higher antimicrobial activity than aqueous extracts (Al-Hashimi, 2012). Moreover, it has been reported that large number of different chemical compounds such as (phenolic compounds and its derivative compounds, the esters of weak acid, fatty acid, terpenes, and others) are presented in ethanolic extracts of spice, and thus these chemical components can affect multiple target sites against the bacterial cells (Burt, 2004; Oonmetta-aree et al., 2006). Similar observations for MIC values, with minor variations, were observed in other studies (Thuille et al., 2003; Tsai et al., 2007; Mostafa et al., 2018).

**Table 3:** The Minimum Inhibitory Concentration (MIC) of plant extracts against the test microorganisms.

Test Strains^∗^	Minimum inhibitory concentration MIC (% w/v)^a^							
	Roselle		Clove		Rosemary		Thyme	
	Ethanol	Water	Ethanol	Water	Ethanol	Water	Ethanol	Water
EC	5	5	2.5	5	5	20	10	5
VP	2.5	5	0.625	2.5	N	N	2.5	10
*PA*	2.5	5	5	10	N	N	N	N
SE	5	10	5	5	2.5	N	N	5
*BC*	5	0.625	2.5	0.313	5	1.25	5	5
*SA*	2.5	2.5	2.5	5	1.25	20	5	2.5
*CA*	N	5	N	N	N	N	20	N

*^a^Values are means of triplicate determination (n = 3). N, no zone of inhibition was found. ^∗^EC, Escherichia coli; VP, Vibrio parahaemolyticus; PA, Pseudomonas aeruginosa; SE, Salmonella enteritidis; BC, Bacillus cereus; SA, Staphylococcus aureus; CA, Candida albicans.*

**FIGURE 2:**
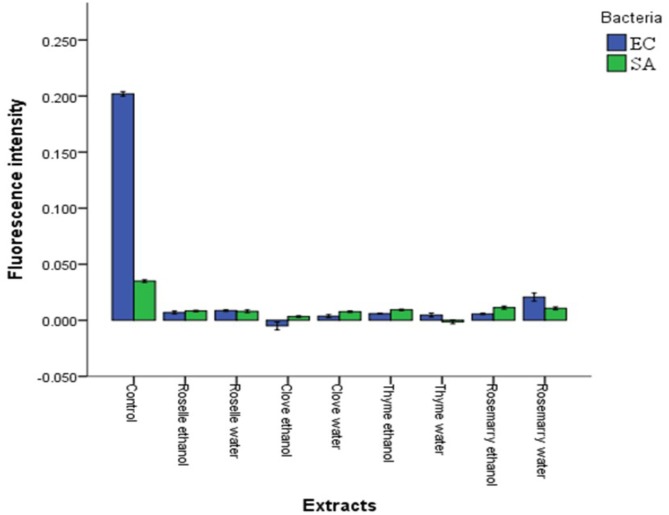
Effects of aqueous and ethanolic extracts of Roselle, Clove, Rosemary, and Thyme on the cytoplasmic pH (pH_in_^*x*^*t*) of *Escherichia coli* (EC) and *Staphylococcus aureus* (SA). Values represent the means of triplicate measurements (*n* = 3). Bars represent the standard deviation.

It is fundamental to develop a better understanding of the antimicrobial mechanism of plant crude extracts on the spoilage and pathogenic microorganisms. Therefore, the effect of plant extracts on cytoplasmic pH_int_ and membrane potential of Gram-positive (SA) and Gram-negative (EC) strains were determined. Significant decreased in cytoplasmic pH_int_ (*P* ≤ 0.05) was observed after addition of plant extracts (**Figure 2**). Ethanolic extracts of clove and water extracts of thyme had the most substantial effect on both SA and EC strains; however, the water extracts of rosemary had the lowest impact compared to other extracts. The changes that occurred in the pH_int_ indicate damage to the bacterial cell membrane (Sanchez et al., 2010). These results are consistent with Lambert et al. (2001) who found that treating SA with oregano essential oil, thymol, and carvacrol causes reduction in the internal pH_int_. Taken as a whole, the fluorescence intensity of the dye inside cells is dependent on the cytoplasmic pH of the cells where low pH results into low fluorescence intensity (Aono et al., 1997; Molina-Gutierrez et al., 2002; Cheng et al., 2015).

**FIGURE 3:**
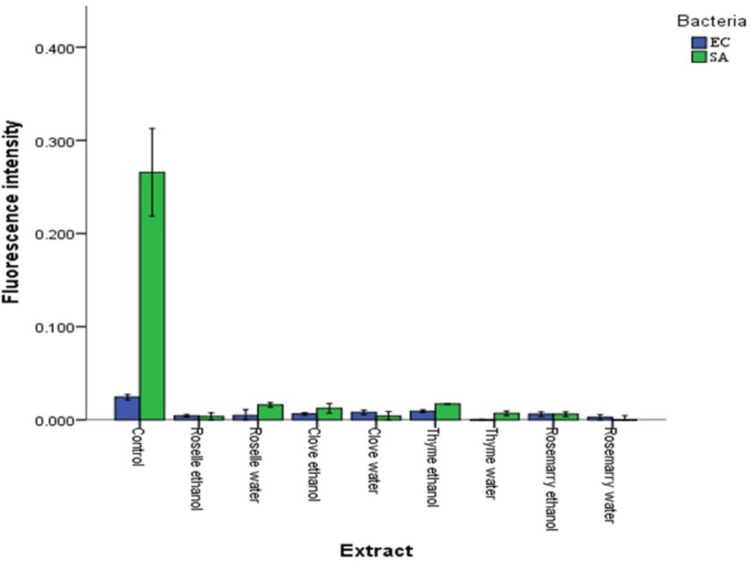
Effects of aqueous and ethanolic extracts of Roselle, Clove, Rosemary, and Thyme on the membrane potentials of *Escherichia coli* (EC) and *Staphylococcus aureus* (SA). Values represent the means of triplicate measurements (*n* = 3). Bars represent the standard deviation.

On the other hand, the changes in the membrane potential of SA and EC were determined after treatment with plant extracts using DiBAC_4_ (3) (a fluorescent dye). Suzuki et al. (2003) stated that the changes in membrane polarization could be measured using DiBAC_4_ (3), a fluorescent membrane potential stain. Our results showed a decrease in the fluorescence intensity of stained cells, which indicated displayed cell membrane hyperpolarization (**Figure 3**). Hyperpolarization has been suggested to be one of the primary indicators of membrane damage of bacteria cells (Vanhauteghem et al., 2013). These findings match with (Sanchez et al., 2010). Being an anionic oxonol dye, DiBAC_4_ (3) has higher permeability and accumulation in cells with polarized membrane potential due to the presence of higher positive charges inside the cytoplasmic membrane (Joux and Lebaron, 2000). On the other side, hyperpolarization results in poor uptake and thus reduce the accumulation of the dye inside the cytoplasmic membrane of the bacteria leading to low fluorescence intensity (Sanchez et al., 2010; Sträuber and Müller, 2010).

Based on the results, it can be concluded that using ultrasound method during extraction effectively improve the extraction yield. Overall, water and ethanolic extracts from selected plants possess antimicrobial activity as they could inhibit the growth of tested food pathogens and spoilage microorganisms. The ethanolic and aqueous extracts of roselle and clove had antimicrobial activity against all tested microorganisms except for the CA that was affected only by ethanolic extracts of clove and thyme. A decrease in cytoplasmic pH (pH_int)_ and cell wall disruption was observed in cells treated with plant extracts, suggesting a possible mechanism of antibacterial action. These findings indicate that the plant extracts tested in this study could be used as natural preservative agents in food to eliminate or control the growth of spoilage and pathogenic microorganisms.

## Author Contributions

FG is a student and was responsible for conducting the antimicrobial assays and contributed to the manuscript revisions mainly materials and methods. JL contributed to the development of the experimental design, data analysis, the manuscript draft, and proofreading. WM contributed to the experimental design, plants extraction, interpreted the results, as well as data analysis, and interpretation. JX and FC carried out the antimicrobial mechanism experiments, helped to write the manuscript. MC and SH were the lead investigators, designed the study, conducted the experiments, supervised the students, drafted the manuscript, and final proofreading.

## Conflict of Interest Statement

The authors declare that the research was conducted in the absence of any commercial or financial relationships that could be construed as a potential conflict of interest.
